# Identifying nurse managers' essential information needs in daily unit operation in perioperative settings

**DOI:** 10.1002/nop2.454

**Published:** 2020-02-08

**Authors:** Eriikka Siirala, Sanna Salanterä, Heljä Lundgrén‐Laine, Laura‐Maria Peltonen, Janne Engblom, Kristiina Junttila

**Affiliations:** ^1^ Department of Nursing Science University of Turku Turku Finland; ^2^ Turku University Hospital Turku Finland; ^3^ Central Finland Health Care District Jyväskylä Finland; ^4^ Department of Mathematics and Statistics University of Turku Turku Finland; ^5^ School of Economics University of Turku Turku Finland; ^6^ Nursing Research Center Helsinki University Hospital University of Helsinki Helsinki Finland

**Keywords:** information needs, nurse managers, nurses, nursing, perioperative settings

## Abstract

**Aim:**

To identify nurse managers' essential information needs in daily unit operation in perioperative settings.

**Design:**

Qualitative and quantitative descriptive design.

**Methods:**

The study consisted of (I) generation of an item pool of potential information needs, (II) assessment of the item pool by an expert panel and (III) confirming the essential information needs of nurse managers in daily unit operation with a survey (*N = *288). Content validity index values were calculated for the assessments by expert panel and in the survey. Internal consistency of the final item pool was explored with Cronbach's alpha. The data were collected from 2011–2015.

**Results:**

During the study process, the number of essential information needs decreased from 92–41. The final item pool consisted of 12 subthemes, and they were categorized into four main themes: patient's care process, surgical procedure, human resources and tangible resources. The findings can be used to create a knowledge map for information system purposes.

## 1 INTRODUCTION

Nurse managers make numerous operative decisions every day without proper and sufficient information (Siirala, Peltonen, Lundgrén‐Laine, Salanterä, & Junttila, 2016) in perioperative settings, especially in operating departments and day surgery units. Nurse managers form the wisdom needed for decision‐making by using data, information and knowledge in daily unit operation (Rowley, 2007). It is possible to partly refine the wisdom needed in decision‐making with existing information systems from different information sources (Gutenstein, Pickering, & Than, 2019). However, present information systems do not support nurse managers in the best possible way, because information is scattered and must be collected from different information sources and not all of the essential information is available. Information systems could better support nurse managers' decision‐making process if the essential information was defined and available at the moment of the decision‐making (Peltonen, Junttila, & Salanterä, 2018; Ranade‐Kharkar et al., 2017).

Knowledge is an immaterial resource. Value, as well as new competitive knowledge, can be created by connecting different information through knowhow (Kianto, Ritala, Spender, & Vanhala, 2014). Studies concerning information processes suggest focusing more on knowledge management and knowledge use studies in addition to focusing on data science. It is recommended that research is done together with end‐users (Gutenstein et al., 2019; McGeorge et al., 2015).

We were interested in identifying nurse managers' essential information needs in perioperative settings to support the creation of a knowledge map of the essential information required to build an information system for them in the future. Knowledge mapping is a method of knowledge management that enables combining large data sets and various information to meet different information needs. (Watthananona & Mingkhwanb, 2012). It is important to identify the information needs of nurse managers in the daily unit operation so that future information systems support their decision‐making in constantly changing situations.

## 2 BACKGROUND

Nurse managers who are responsible for the daily unit operations in perioperative settings are accountable for achieving the goals that have been set up on the strategic level (Baker et al., 2012). Nurse managers are working under constant pressure from the strategic level and also from the clinical level. Studies have mainly focused on the strategic planning and scheduling of the operating departments’ resources (Gür & Eren, 2018). Traditionally, the tactical planning of services starts by allocating operating room time for different medical specialties. The necessary resources of nursing care are planned after the allocation of time slots for the medical specialties (Di Martinelly, Baptiste, & Maknoon, 2014). The operative planning of the unit is usually performed about 2 days before a planned operation (e.g. nurse assignment and scheduling surgical procedures; Dexter, Shi, & Epstein, 2012; Levine & Dunn, 2015). The operative planning is one major task of nurse managers. This planning has a notable influence on operating departments' productivity (Levine & Dunn, 2015; Peltokorpi, 2011).

**Figure 1:**
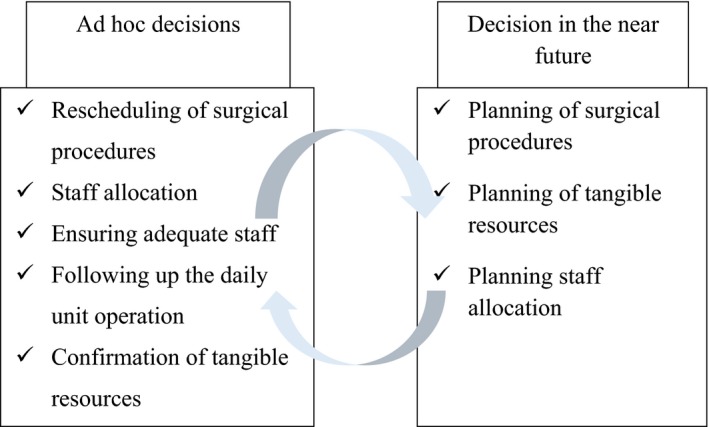
Framework of the decisions made by nurse managers in daily unit operation. The framework is modified for the purpose of this study from a previously published study (Siirala et al., 2016)

A framework of the operational decisions made by nurse managers in daily unit operation in perioperative settings has previously been defined. In the framework, decisions made by a nurse manager are divided into three themes that are “ad hoc decisions,” “decisions in near future” and “long‐term decisions” (Siirala et al., 2016). Long‐term decisions are not bound to the daily unit operation as decision‐making in daily unit operation usually happens simultaneously. Therefore, we focused on the themes “ad hoc decisions” and “decisions in near future” (Figure 1). “Ad hoc decisions” mean that the nurse manager needs to constantly be aware what happens at the department. She/he is susceptible for sudden changes and ready to change the daily unit operation into new direction. “Decisions in the near future” are focusing on the next day's activities at the department. Decision‐making in this theme is vulnerable for interruptions due to the ad hoc decisions. Time is limited for decision‐making; thus, the information needs to be available (Siirala et al., 2016).

Challenges arise when the needed information is not available in the constantly changing situations. Different medical specialties and elective and emergency operations are often performed simultaneously in the same operating department. Daily unit operations are characterized by constant uncertainty. Nurse managers need to predict and anticipate the uncertainty caused by emergency operations (Cardoen, Demeulemeester, & Beliën, 2010; Wiyartanti et al., 2015). Daily unit operation is challenging because the information needed in decision‐making is scattered, and a general overview of the situation of the unit is difficult to form (Peltonen, Junttila, et al., 2018). For example, the assignment of nurses in the operating rooms is usually made manually and then moved to an information system (Levine & Dunn, 2015). Multiple entries of the same data are susceptible to human error. Knowledge of the special characteristics of operating rooms, the knowhow of nurses and of broken equipment is not kept on a database, and nurse managers have to remember the details. But it is difficult to remember all the information needed in daily unit operation.

A lack of information in daily unit operation changes the scheduled of planned operations. Sudden changes during surgical procedures are common, and together with rescheduling the daily unit operation, the changes affect the stress experienced by nurses (Minnick, Donaghey, Slagle, & Weinger, 2012). Currently, nurse managers are dissatisfied with available information systems that support their work (Peltonen, Junttila, et al., 2018), which may impede efficient work and decrease job satisfaction.

Due to our interest in identifying the information needs of nurse managers in perioperative settings, we explored potential instruments or tools to be used. An instrument measuring the information needs of nurse managers in intensive care units (ICUs) already exists (Lundgrén‐Laine et al., 2013). An instrument is usually developed for a specific context and may not fit other contexts (DeVellis, 2012). Due to the characteristics of perioperative settings, the above‐mentioned instrument for exploring nurse managers' essential information needs in ICUs is only partly suitable in perioperative settings. Therefore, we first gathered information needs of nurse managers in perioperative settings with a think aloud method and then determined the relevance of these needs to nurse managers with a survey method.

This study aimed to identify nurse managers' essential information needs in daily unit operation in perioperative settings (operating departments and day surgery units). The goal was to identify essential information for the creation of a knowledge map. The knowledge map can be used when developing the content for an information system in a perioperative setting. The research question was as follows: “What are the nurse managers' essential information needs in daily unit operation in perioperative settings?”

## 3 THE STUDY

### 3.1 Design

**Figure 2:**
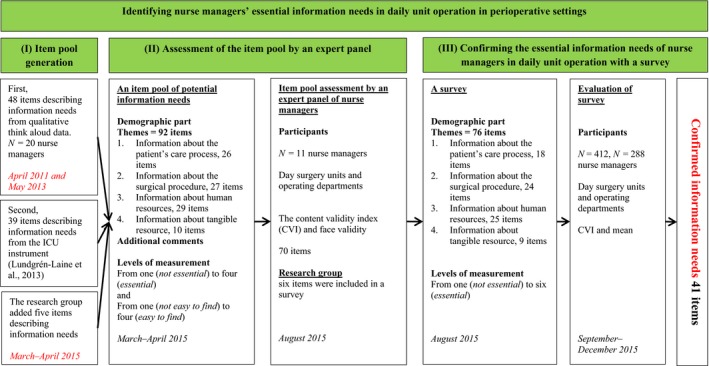
A summary of the study design

We used both qualitative and quantitative descriptive methods. The study followed three phases: (I) generation of an item pool of potential information needs; (II) assessment of the item pool by an expert panel; and (III) confirming essential information needs of nurse managers in daily unit operation with a survey. (DeVellis, 2012.) The study design is presented in Figure 2.

### 3.2 Participants

The participants in (I) the item pool generation, (II) in the expert panel and (III) in the survey were nursing professionals who were in charge of daily unit operations during office hours (from 8 a.m.–4 p.m.) in perioperative settings. The term *nurse manager* is here used for all the nursing professionals, that is charge nurses, assistant nurse managers and administrative nurse managers who were in charge of the daily unit operations in an unit, that is an operating department or a day surgery unit. In Finland, the administrative nurse managers do not usually participate in direct patient care and the unit is mainly run from an office. The managerial experience was not focused in our study. Instead, we wanted to have participants from novice to expert to capture all essential information needs.

In first phase of the study (I), that is item pool generation, qualitative data were partly used. This data consisted of nurse managers' (*N = *20) think aloud sessions. In the item pool assessment (phase II), an expert panel of 11 nurse managers was used. The participants in the expert panel were working in one operating department and in one day surgery unit. The PI contacted the administrative nurse managers of the two departments to identify and recruit eligible participants when the study approval was obtained. The survey to confirm the essential information needs (phase III) was conducted in all five university hospitals in Finland and in a random selection of five public‐funded secondary‐level hospitals. In Finland, public health services are divided into primary health care and specialized medical care. Specialized medical care refers to secondary and tertiary health care. University teaching hospitals offer tertiary‐level and central hospitals the secondary‐level services in medical specialities (Ministry of Social Affairs & Health, 2019). The sample consisted of 412 eligible participants of which 288 (70%) responded to the survey.

### 3.3 Data collection and analysis

#### 3.3.1 Item pool generation

The item pool generation used two data sources: the first was based on 20 nurse managers who were shadowed while they described their decisions and information needs with the think aloud method (Van Someren, Barnard, & Sandberg, 1994). This data were collected in 2011 and 2013 in operating departments and day surgery units in two university hospitals. Decisions made by the nurse managers have been reported previously (Siirala et al., 2016), and their information needs are reported here. The second data source used for the item pool generation was a previously developed instrument for exploring ICU nurse managers' information needs (Lundgrén‐Laine et al., 2013). Permission to use the instrument was granted by the copyright holder.

*First*, the think aloud data consisted of 63 hr of talking. These data were transcribed into Microsoft Word by the Primary Investigator (PI). Every single information need together with a decision made by the nurse manager was highlighted from the text. Then, the highlighted texts were moved to a Microsoft Excel sheet. We used thematic analysis when formulating the information needs from the think aloud data (Braun & Clarke, 2006). The identified items of information needs were categorized into subthemes, which were further categorized into main themes. An example of the thematic analysis process is presented in Table 1.

**Table 1:** An example of the thematic analysis of the information needs: The ongoing situation at the department and the information needs

Free text	The situation at the department	The information need	Subtheme	Main theme
“There are two gastro emergency operations but unfortunately we have no room available” (P)	An emergency operation is on the waiting list	Specialty	Surgical procedures	Information concerning decisions about the surgical procedure
		Available operating rooms	Operating rooms	Information concerning decisions about tangible resources
		The urgency of the operation	Scheduling surgical procedures	Information concerning decisions about the surgical procedure
“The patient is allowed to be on the waiting list. If it is possible to operate outside the office hours then the operation will be done. If it is not possible, then they will be operated on tomorrow. At the moment, we are not able to make a plan, because all the operating room are booked. Does he [the surgery] know about it? Although we have a surgeon available, we do not have an operating room available. …. If we get an ICU permit … [the nurse asks about the operating table] Yes, the operating table can manage heavy patients but not very much turning patient's side by side. We will operate either in bed or on the operating table.” (I)	An emergency operation is on the waiting list.	Operations outside office hours	Scheduling the surgical procedures	Information concerning decisions about the surgical procedure
		The urgency of the operation	Scheduling the surgical procedures	Information concerning decisions about the surgical procedure
		Available operating rooms	Operating rooms	Information concerning decisions about tangible resources
		Surgeon	Surgeons and anaesthesiologists	Information concerning decisions about human resources
		The follow‐up department	Postanaesthesia care unit	Information concerning decisions about the surgical procedure
		Patient information	Background information on the patient	Information concerning decisions about the patient's care process
		The equipment and materials in the operating room	Operating rooms	Information concerning decisions about tangible resource
		The surgical position	Surgical procedures	Information concerning decisions about the surgical procedure
“I need to check the ongoing operations [looks at the enterprise resource planning] due to the reason that we have many nurses who go home at 2 p.m. We need to know what we are able to operate after that. It seems that three operations are now beginning. These operations will be done during the office hours but we also have one huge operation that might last longer. I need a team to continue after office hours. We also have three operations on the waiting list, but I guess we are only able to do one and these two might be cancelled if we do not have any voluntary nurses working overtime. Once we know this, we will know if we need to cancel these. In addition, we have one emergency operation outside the department that is not in the ERP yet. We need to reserve one nurse to do that.” (Q)	Checking the daily unit operations	The duration of the operations	Scheduling the surgical procedures	Information concerning decisions about the surgical procedure
		The duration of the shift	The shifts of the staff	Information concerning decisions about human resources
		The number of teams on call	The shifts of the staff	Information concerning decisions about human resources
		The starting time of the operation	Scheduling the surgical procedures	Information concerning decisions about the surgical procedure
		Overtime working possibilities	The shifts of the staff	Information concerning decisions about human resources
		Operations outside the operating department	Scheduling the surgical procedures	Information concerning decisions about the surgical procedure

*Second*, the instrument measuring ICU nurse managers' information needs consisted of 122 items (Lundgrén‐Laine et al., 2013). Their relevance was assessed by the research group and, based on consensus, the items were either rejected or accepted in the item pool. The research group consisted of nurse scientists, who also have professional experience in perioperative nursing or ICU nursing and a statistical scientist. The item pool generation is presented in Table 2.

**Table 2:** Item pool generation

Themes and subthemes	The ICU instrument (Lundgrén‐Laine et al., 2013)	The information needs derived from qualitative think aloud data (Siirala et al., 2016)	Items added by the research group
Information concerning decisions about the patient's care process: 26 items			
Background information on the patient (1–10)	6	2	2
Patient needs for isolation (11–12)	2	—	—
Patient needs for special treatment (13–20)	3	5	—
Follow‐up treatment (21–26)	4	2	—
Information concerning decisions about the surgical procedure: 27 items			
Surgical procedures (27–39)	3	8	2
Scheduling the surgical procedures (40–48)	2	7	—
Postanaesthesia care unit (49–53)	—	5	—
Information concerning decisions about human resources: 29 items			
The shifts of the staff (54–65)	9	3	—
The assignment of staff (66–73)	6	2	—
The job orientation of new employees and nurse students (74–76)	2	1	—
Surgeons and anaesthesiologists (77–82)	—	5	1
Information concerning decisions about tangible resources: 10 items			
Operating rooms (83–85)	—	3	—
The instruments, materials, and equipment of the operating department (86–92)	2	5	—
Total	39	48	5

#### 3.3.2 Item pool assessment by an expert panel of nurse managers

Identified items were assessed by an expert panel after the item pool generation. The expert panel included nurse managers (*N = *11) from one operating department and one day surgery unit. The participants met in groups of two to three persons with the PI (Grant & Davis, 1997). Each face‐to‐face panel session lasted 1 hr and was arranged during the participants' shift. Items were assessed from two perspectives: the relevance and the availability of the information. The assessment was done with a four‐point Likert scale; the relevance of information ranged from one (*not essential*) to four (*essential*) and the availability of information ranged from one (*not easy to find*) to four (*easy to find*). Next, the PI discussed the relevance of each item with the panel participants.

The item‐level content validity index (CVI) values were calculated to explore the relevance of each item. The responses ascribing a value of three or four were summed up. Then, the number of responses was divided by the total number of participants (Polit & Beck, 2006). All the items that had a score 0.80 or above were kept in the item pool.

We decided to exclude the analysis of the availability of items. The panel sessions clearly revealed that the experts found it confusing to assess two different aspects of each item in the item pool (relevance and availability). Also, the assessment form was very long (five A4 pages).

#### 3.3.3 Confirming the essential information needs of nurse managers

Next, the essential information needs identified in phase II were confirmed by sending a survey to 412 eligible participants in Finland. The estimated sample size was based on the calculation of having five participants to one item (Gorsuch, 1983). The administrative nurse manager of each participating unit was informed about the study. Then, the administrative nurse manager delivered an envelope with the survey in it to potential participants in his or her unit. Participation was voluntary. The administrative nurse manager from each department collected the surveys and returned them to the PI by mail. The participants were all nursing professionals, that is charge nurses, assistant nurse managers and administrative nurse managers. When a charge nurse is responsible for the unit, she/he needs to have the same access to relevant information as the administrative nurse manager has. To make the reporting more fluent, we decided to use the term “nurse manager” for all participants in this article.

A six‐point Likert scale was used to measure the relevance of the items. The scores ranged from one (*not essential*) to six (*essential*). The six‐point rating scale excluded an ambivalent middle point. Thus, the Likert scale was changed from the four‐point scale used in phase II to a six‐point scale in phase III. The change made it possible to gather more detailed information about the relevancy of the information needs.

SAS version 9.4 for Windows was used for calculating participant demographics. The data were manually transcribed from the survey to Microsoft Excel and then moved to SAS. The CVI value for each item in the item pool was calculated (Polit & Beck, 2006) with the Excel program. The values were estimated in the same way as in the previous study phase (II). The values were calculated so that every item that was valued five or six was added and the sum was divided by the number of respondents. For example, 200 respondents gave a value of either five or six; thus, 200 was divided by the total number of participants (288), leading to the result of 0.69.

Although this study did not aim at instrument development, we wanted to ensure the internal consistency of the final item pool by calculating Cronbach's alpha (Cronbach & Gleser, 1957). Values above .70 are recommended for a reliable instrument.

Finland is a bilingual country with Finnish and Swedish as official languages. The item pool generation and assessment (phases I and II) were done in Finnish, and then, the survey was translated into Swedish. The content validity of the Swedish version was assessed by three Swedish‐speaking experts in perioperative nursing and/or nursing science. One of the experts had Swedish as a mother tongue; the two others were bilingual (Finnish and Swedish).

### 3.4 The ethical procedure

The study was conducted in accordance with the Finnish National Board on Research Integrity (2012). Research Ethics Committee approval and permission to conduct the study were obtained from each hospital's authorities. The study followed the principles of respecting the voluntariness, right to refuse and autonomy of research subjects, avoiding any harm to the subjects, and securing the privacy of subjects in all phases of the study.

## 4 RESULTS

### 4.1 Item pool generation

In the item pool generation (I), 48 items were included from the think aloud data and 39 items were derived from the ICU instrument. Items in the ICU instrument were rejected by the research group if they were only ICU‐specific. Otherwise, items were included in the item pool. In addition, the research group added five relevant items to the item pool based on their clinical expertise. Finally, there were 92 items.

The 92 items were categorized into 13 subthemes and further into four main themes. The first main theme, “information concerning decisions about the patient's care process,” consisted of background information on the patient, patient needs for isolation, patient needs for special treatment and follow‐up treatment. The second main theme, “information concerning decisions about the surgical procedure,” consisted of surgical procedures, scheduling the surgical procedures and the postanaesthesia care unit. The third main theme, “information concerning decisions about human resources,” consisted of the shifts of the staff, the assignment of the staff, the job orientation of new employees and nurse students, surgeons and anaesthesiologists. The last main theme, “information concerning decisions about tangible resources,” consisted of the operating rooms, the instruments, materials and equipment of the operating department (Table 2).

### 4.2 Item pool assessment by an expert panel of nurse managers

The number of nurse managers (*N = *11) in the expert panel was seen as acceptable when calculating the CVI on an item level. According to the CVI calculation, of the initial 92 items, 70 were kept in the item pool. The research group included six additional items with CVI values under 0.80 in the pool due to their clinical relevance. The main themes of the item pool did not change after the expert panel assessment. One subtheme, “follow‐up treatment,” was left out. One item was moved to another subtheme. After study phase II, the item pool consisted of 76 items.

### 4.3 Confirming the essential information needs of nurse managers

The survey with 76 items was sent to 52 units. Of the 412 eligible participants, 288 answered to the survey. The response rate was 70%. The participants returned the questionnaires to the PI by mail. The demographic information of the participants is presented in Table 3. Most (85%) of the participants were RNs (with a bachelor's degree). The rest of the participants also had a master's degree. The participants were mainly (80%) working in university hospitals; the rest worked in secondary‐level hospitals. Of the participants, 74% informed that emergency operations are done at their department. In addition, operations outside the department were done in most departments (76%).

**Table 3:** Demographic information about the participants

Demographic information	Mean	Range
Work experience as a nurse in an operating department	19 years	1.5–40 years
Work experience as a nurse manager	8 years	0–36 years
The frequency of acting as a nurse manager during office hours in an operating department	73 shifts/year	0–275 shifts
The number of operating rooms in the department	8 ORs	0–28 ORs1
The number of beds in the postanaesthesia care unit	11 beds	0–50 beds^a^

a Value 0 refers to an anaesthesia department which offers nursing services to other units in a hospital (e.g. operating department) thus does not have a physical department of its own.

The CVI values of the items showed that 41 items were essential for nurse managers and had a CVI value equal to or over 0.80. Thus, the final item pool consisted of 41 items. The four main themes remained same as in previous study phases. Detailed information about the confirmed essential information needs is presented in Table 4.

**Table 4:** Confirmed information needs (*N* = 41)

Information concerning decisions about the patient's care process	CVI
Background information on the patient	
1. Information about the diagnosis that affects the patient's care process	0.84
2. Information from complications caused by earlier surgical or anaesthesia procedures	0.88
3. Information about the patient's planned time of surgery	0.82
4. Information about the patient's divergent laboratory values (e.g. INR, Hb in connection with the local anaesthesia)	0.93
5. Information about the blood samples to be taken for surgery or anaesthesia	0.80
6. Information about the patient's surgery and anaesthesia preparation	0.82
Patient needs for isolation	
7. Information about the need for isolation (e.g. MRSA, VRE)	0.96
8. Information about the form of isolation (e.g. infection isolation, touch isolation)	0.96
Patient needs for special treatment	
9. Information about the length of the *nil per os* time	0.94

Information concerning decisions about the surgical procedure	CVI
Surgical procedures	
10. Information about the urgency	0.95
11. Information about the patient's surgical procedure	0.98
12. Information about the sequence of surgical procedures	0.93
13. Information about the placement of the patient's operating room	0.89
14. Information about the patient's anaesthesia form	0.91
15. Information about the patient's position during the operation	0.83
16. Information about the estimated challenges (e.g. an old fracture, difficult intubation) there will be in the operation or administering anaesthesia	0.92
17. Information about the phase of an ongoing operation in an operating room	0.88
18. Information about a complication during the operation	0.87
Scheduling the surgical procedures	
19. Information about the number of planned operations	0.84
20. Information about the number of possible emergency operations	0.84
21. Information about the starting time and length of an operation	0.80
Postanaesthesia care unit	
22. Information about the patient's follow‐up in the operating room after an operation (e.g. need for an ICU)	0.84

Information concerning decisions about human resources	CVI
The shifts of the staff	
23. Information about the shifts of the nursing staff	0.97
24. Information about the full‐complement strength of the nursing staff on a shift	0.97
25. Information about the staff required during the operation phases in an operating room	0.90
26. Information about the special characteristics of the shift (a standby shift)	0.95
27. Information about nurses working overtime	0.95
The assignment of staff	
28. Information about a nurse's assignment during a shift	0.82
29. Information about detachable staff resources	0.91
30. Information about the knowhow of the nursing staff (e.g. an anaesthesia nurse, special knowhow, language skills)	0.90
The job orientation of new employees and nurse students	
31. Information about nurses in the orientation	0.83
Surgeons and anaesthesiologists	
32. Information about the surgeon	0.91
33. Information about the surgeon's overlapping operations	0.93
34. Information about the anaesthesiologist	0.89
35. Information about the anaesthesiologist's overlapping operations	0.89

Information concerning decisions about tangible resources	CVI
Operating rooms	
36. Information about the operating rooms in which there is an ongoing operation	0.94
37. Information about the fixed materials and equipment of operating rooms (e.g. the operating table and imaging possibilities)	0.87
38. Information about the relevance of operating room equipping in regard to a potential procedure	0.88
The instruments, materials, equipment of the operating department	
39. Information about the adequacy of the instruments in regard to conducting several operations simultaneously	0.88
40. Information about the urgency of the instruments to measure (the number and adequacy)	0.84
41. Information about the instruments required for special operations	0.84

The confirmed information needs (*N = *41 items) formed the following main themes: patient's care process (subthemes: “background information on the patient,” “patient needs for isolation,” “patient needs for special treatment,” *N = *9 items), surgical procedures (subthemes: “surgical procedures,” “scheduling the surgical procedures,” “postaneasthesia care unit,” *N = *13 items), human resources (subthemes: “the shifts of the staff,” “the assignment of staff,” “the job orientation of new employees and nurse students,” “surgeons and anaesthesiologists,” *N = *13 items) and tangible resources (subtheme: “operating rooms,” “the instruments, materials, equipment of the operating department,” *N = *6 items). Cronbach's alpha values of the main themes are presented in Table 5. They varied from .80–.88. The result indicated good internal consistency in every main theme.

**Table 5:** The Cronbach's alpha for the main themes (*N* = 41)

Standardized variables for the main themes	Alpha
Information concerning decisions about the patient's care process	.80
Information concerning decisions about the surgical procedure	.81
Information concerning decisions about human resources	.88
Information concerning decisions about tangible resources	.86

## 5 DISCUSSION

This study aimed to identify nurse managers' essential information needs in daily unit operation in perioperative settings (operating departments and day surgery units). The information will be used when creating a knowledge map (Watthananona & Mingkhwanb, 2012). A knowledge map also helps to measure and personify the information needs in future. It will enhance the achievement of objectives set for the daily unit operation at a strategic level. In a later phase, the knowledge map can support the evaluation of hospital performance by indicating the outcomes that should be evaluated in daily care (Markazi‐Moghaddam et al., 2016). Based on our prior knowledge, instruments have been developed to measure the information needs of nurse managers in acute healthcare settings but not in operating departments and day surgery units (Lundgrén‐Laine et al., 2013; Peltonen, Siirala, et al., 2018). This study identified nurse managers' essential information needs in daily unit operation in perioperative settings.

The study phases included (I) item pool generation, (II) assessment of item pool by an expert panel and (III) confirmation of the essential information needs of nurse managers (i.e. the content of the item pool) with a survey. During the process and by using CVI values as criteria, the initial number of items in the item pool decreased from 92–41. The final 41 items, categorized into 12 subthemes and four main themes, can be used when creating a knowledge map for the content of an information system in perioperative settings. The information systems that are currently used do not support the nurse managers' decision‐making sufficiently. Information is scattered and not easy to find during the daily unit operation (Peltonen, Junttila, et al., 2018). In ideal situation, nurse manager should have a single dashboard, where the essential information needs are presented. Together with artificial intelligence, the information system could support nurse managers' decision‐making with relevant information.

In the study phase II, the content validity of item pool was assessed by an expert panel whose members were familiar with the content of nurse managers' tasks (DeVellis, 2012). The discussions with the panel participants did not bring forward any more detailed information needs that should have been included in the item pool. After the item pool assessment, the CVI value of the subtheme “follow‐up treatment” (information concerning decisions about the patient's care process) was not relevant to daily unit operation. The “follow‐up treatment” covered six items that focused on a postoperative period in a ward. An earlier study pointed out that nurse managers are also responsible for dealing with glitches in interdepartmental processes (Baker et al., 2012). However, the respondents clearly indicated that they were not willing to take responsibility for the processes in other units that can hamper the overall care process of a patient and the smooth flow of the operation. Although the CVI values after the item pool assessment indicated 70 essential information needs, the research group included six additional items in the pool due to their clinical relevance.

The demographic information of the participants in the survey (phase III) showed that nurse managers have, on average, almost 20 years of experience of nursing in perioperative settings. A previous study conducted in operating departments pointed out that also nurse managers are well experienced based on their years in management or leadership role (Baker et al., 2012; Sherman, Patterson, Avitable, & Dahle, 2014). Sherman et al. (2014) reported that 72.2% (*N = *256) of the nurse *leaders* in their study had more than ten years of experience of the management role. In Baker's (2012) study, 60% (*N = *17) of the respondents had more than 5 years of experience of management. This was also seen in our study where nurse managers were experienced (mean 8 years). Our study's results strengthen earlier studies that observed that operating departments and day surgery units are complex environments. There are many different specialists working at the same time with both elective and on‐call operations (Cardoen et al., 2010; Wiyartanti et al., 2015). It is important to find out new ways of organizing the work for the next generation.

In study phase III, the CVI values (Polit & Beck, 2006) pointed out that more than half (*N = *41) of the assessed information needs (*N = *76) were essential for all nurse managers in daily unit operation. The confirmed 41 information needs should be considered when developing the content of information systems intended to support nurse managers in operating departments. In addition, the CVI values revealed information needs that were not assessed to be relevant.

In study phase III, the item pool had a subtheme named “patient needs for special treatment” (under the theme “information concerning decisions about the patient's care process”) which included items describing the cultural background of the patient, his or her special needs (e.g. hearing loss), previously experienced pain, information about relatives, the hoped‐for operation time and his or her living will. CVI values of the aforementioned information needs pointed out that they were not essential for nurse managers. Information needs focusing on patient needs for special treatment are related to the pre‐operative phase and actual care process of the surgical procedure and should have already been considered before the final decision about the operation. The length of the *nil per os* time was thought to be essential for the nurse manager and was included in the final item pool.

The purpose of this study was to capture the essential information needs for the creation of a knowledge map that can be used in building an information system in daily unit operation rather than to develop an instrument. So, instead of using factor analysis to confirm construct validity in study phase III, the essential information needs were confirmed with the CVI (Polit & Beck, 2006). A result from factor analysis may have shown the construct validity of the item pool but may also have left out relevant items needed for knowledge mapping. However, we wanted to ensure the internal consistency of the final item pool by calculating Cronbach's alpha values for the four main themes. The values indicate that the final 41 item pool captures the phenomena of nurse managers' information needs in daily unit operation.

The identified four main themes are closely related to the key areas that nurse managers focus their tasks on in daily unit operation (Siirala et al., 2016). Our findings are consistent with a published study about nurse managers' responsibilities (Levine & Dunn, 2015). However, our study focused more on the concrete level of information that is needed in management. The results of this study, together with an earlier published study (Siirala et al., 2016), can be used in defining further the content of the nurse managers' tasks and in creating a knowledge map in the future. A previous study (Gutenstein et al., 2019) concluded that the end‐users are essential when creating new information systems. Our study has taken nurse managers into consideration as end‐users.

The planning of daily unit operation usually happens a few days in advance (Dexter et al., 2012; Levine & Dunn, 2015), and successful planning is connected to smooth unit operation and its productivity (Levine & Dunn, 2015; Peltokorpi, 2011). Our study aims to reduce the manual work of the nurse manager by revealing the essential information that can be stored in an information system (Levine & Dunn, 2015).

## 6 STRENGTHS AND LIMITATIONS

There are some strengths and limitations in this study. One strength was that this study was done with participants who are experts and familiar in the management of perioperative settings. Developing a new information system to support daily unit operation should be done together with those who are the possible users (Gutenstein et al., 2019; McGeorge et al., 2015). Therefore, the participants were familiar with the topic and the content of the survey was not sensitive. For this reason, the Likert scale used did not have a middle point (Tsang, 2012).

Our survey aimed to identify essential information needs that are relevant in ad hoc decision‐making in perioperative settings. Our aim was not to develop or validate an instrument, and this might limit the further use of it. We conducted a paper‐based survey instead of an electronic version. Even though an electronic version has many advantages, like fast deployment and low costs (McPeake, Bateson, & O'Neill, 2014), the paper format proved to be a good choice based on the response rate (70%). The data from the survey were transported manually to the SAS program which always is vulnerable for mistakes. Mistakes were tried to hinder by conducting the work in an office, which is only for the researchers for working in peace. The translation of the survey into Swedish in study phase III was successful as the answers in both versions (Swedish and Finnish) were consistent. The data were collected in three phases from 2011–2015. During these years, and after, there may have been some organizational changes that can affect the knowledge need.

Traditionally, anaesthesiologists, different surgical specialists and nursing staff work together in the operating departments and day surgery units (Di Martinelly et al., 2014). In our study, we only focused on nurse managers' information needs, not on those of the surgeons or anaesthesiologists. In addition, our study participants were working in nurse managers' positions during office hours. We did not focus on the nurses who are working in charge position outside the office hours (from 4 p.m.–8 a.m.). There might be limitations regarding the information needs due to that perspective.

## 7 CONCLUSION

This study identified the essential information needs of nurse managers in daily unit operation in perioperative settings (operating departments and day surgery units). The four main themes (including 41 items) showed good internal consistency. The CVI values pointed out that nurse managers have several common essential information needs in daily unit operation regardless of their work environment. The confirmed information needs can be used to create a knowledge map to be used in building an information system which better supports nurse managers in perioperative settings.

## CONFLICT OF INTEREST

The authors declare no conflict of interest.

## AUTHOR CONTRIBUTIONS

Eriikka Siirala: Substantial contribution for the conception and design of the study, the acquisition, data analysis and interpretation and manuscript writing. Sanna Salanterä, Heljä Lundgrén‐Laine, Laura‐Maria Peltonen and Kristiina Junttila: Conception and design of the study and data interpretation. Janne Engblom: Data acquisition and data analysis and interpretation. All have critically revised the manuscript for important intellectual content. All the named authors have given final approval of the version to be published.
